# Regulating voltage-gated ion channels with nanobodies

**DOI:** 10.1038/s41467-022-35027-5

**Published:** 2022-12-09

**Authors:** Declan Manning, L. Fernando Santana

**Affiliations:** Department of Physiology and Membrane Biology, University of California, Davis, One Shields Avenue, Davis, CA USA

**Keywords:** Calcium channels, Permeation and transport, Ion channels in the nervous system

## Abstract

In this work, Morgenstern and colleagues describe an approach involving functionalized nanobodies which decrease the activity of voltage-gated Ca^2+^ channels associated with β_1_ subunits and promote their removal from the surface membrane of neurons and muscle.

## Voltage-gated Ca^2+^ channels are composed of multiple subunits and are broadly expressed in excitable cells

Voltage-gated Ca^2+^ (Ca_V_) channels are broadly expressed in excitable cells throughout the body, where they regulate multiple physiological processes, including cardiac, skeletal, and smooth muscle contraction as well as neuronal excitability, neurotransmitter release, and gene expression. Accordingly, Ca_V_ channels are a major target for the treatment of cardiovascular and neurological disease. Morgenstern and colleagues^1^ describe an approach for controlling the function of Ca_V_ channels with a specific molecular composition.

Ca_V_ channels are formed by one of ten pore-forming α_1_ subunits, which largely determine their conductive properties. Four of these subunits contribute to L-type Ca^2+^ channels (Ca_V_1.1-4), while R-, N-, and P/Q-type channels (Ca_V_2.1-4) are composed of their own distinct α_1_ subunits.

The Ca_V_1 and Ca_V_2 subfamilies associate with a range of auxiliary subunits: β, encoded by *CACNB1-4*; α_2_δ, encoded by *CACNA2D1-4*; and γ, encoded by *CACNG1-8*^2,3^. α_1_ subunits form a strong interface with β subunits at the intracellular α-interaction domain of the channel^4,5^. It is believed that Ca_V_1 and Ca_V_2 channels all assemble with a single β subunit, which serves to promote membrane expression and set channel voltage-dependencies. Expression of β_1-4_ subunits has been detected in the brain. β_1_ is also expressed in skeletal muscle, whilst β_2_ subunits are expressed in heart, lung, and smooth muscle. Finally, β_3_ subunits are expressed highly in smooth muscle.

The β subunit prevents ubiquitination of the α_1_/β channel complex, increasing membrane channel density and whole-cell current magnitude^6^. β subunits also confer diverse biophysical properties to the mature Ca_V_ channels, enabling G protein regulation, setting rates of channel activation and inactivation, and fine-tuning voltage dependency^7^. β subunits also serve as sites for post-translational modification and protein-protein interactions. For example, palmitoylation allows β_2a_ to embed into the plasma membrane, slowing the rate of channel inactivation^8^. More recently, adrenergic stimulation of Ca_V_1.2 in the heart was shown to involve an interaction between β_2a_ and the Rad G protein, which becomes phosphorylated by protein kinase A to relieve its constitutive inhibition of Ca_V_1.2^9^.

Most Ca_V_ channel agonists and antagonists work by binding to their α_1_ subunits. However, because of the broad expression of Ca_V_ channels, this pharmacological approach does not afford precise, tissue-specific regulation of Ca^2+^ entry. Gene ablation or siRNA-mediated protein knockdown of β or other subunits approaches could circumvent these limitations, but the interpretation of these experiments is confounded when there are multiple auxiliary subunit isoforms expressed in a cell, some of which have partially overlapping functions.

## Nanobodies target ion channels of specific composition with precision

Here, Morgenstern et al.^1^ describe an elegant and highly effective strategy to decrease the functional impact of β_1_-associated Ca_V_ channels using nanobodies.

Nanobodies are recombinant antigen binding fragments, and their small size and folding properties enhance their stability inside of live cells, where they can be utilized as “intrabodies”^10^. Morgenstern et al.^1^ demonstrate how Ca_V_ channels may be targeted with functionalized nanobodies, to precisely inhibit channels comprising of specific β subunit isoforms.

In their previous work, Morgenstern et al.^11^ demonstrated a Ca_V_β-targeted nanobody (nb.F3) inhibits Ca_V_1/2 channels by initiating their redistribution into endosomes. This nanobody-delivered ubiquitination machinery (Ca_V_-aβlator) functions as an effective inhibitor of Ca_V_ channels. In this present work, Morgenstern et al.^1^ reveal a refined inhibitor specifically targeted to β_1_-associated Ca_V_ channels (Chisel-1).

Briefly, the authors identified a nanobody (nb.E8) which selectively binds the Ca_V_β_1_ SH3 domain and inhibits Ca_V_β_1_-associated voltage-gated Ca_V_ channels by decreasing open probability and increasing their rate of channel inactivation. Interestingly, nb.E8 also decreases channel activity by reducing channel surface density.

Functionalizing nb.E8 with the Nedd4L HECT domain yielded Chisel-1, which eliminated current through Ca_V_β_1_-reconstituted Ca_V_1/Ca_V_2 and native Ca_V_1.1 channels in skeletal muscle. Chisel-1 also decreased depolarization-induced Ca^2+^ entry and excitation-transcription coupling in hippocampal neurons. Notably, Chisel-1 was ineffective against Ca_V_β_2_-associated Ca_V_1.2 channels in cardiomyocytes, underscoring its specificity. In a therapeutic setting, genetically-encoded inhibitors like Ca_V_-aβlator and Chisel-1 could be selectively expressed within cells of interest, potentially bypassing the off-target effects produced by many traditional Ca_V_ inhibitors.

## Nanobodies could reveal important aspects of ion channel organization and function

The findings by Morgenstern et al.^1^ raise multiple intriguing questions. For example, how does binding of the nb.E8 nanobody to Ca_V_β_1_, independent of ubiquitin ligase conjugation, act to reduce the membrane surface density of Ca_V_2.2 channels? Does the reduction in channel activity in the presence of nb.E8 prime the channel for endocytosis? Also, Ca_V_ channels form clusters in the surface membrane of neurons and muscle^12,13^. Recent studies indicate that ion channels involved in cooperative signaling cascades co-cluster. This raises the question of whether Chisel-1-bound channels are removed individually within a cluster or if the binding of a subset of channels destines the entire cluster for removal? The latter mechanism would suggest an amplification mechanism which could impact on clustered channels. Future studies should investigate these questions.
